# Pilot of a bowel habit assessment tool to enable early identification of *Clostridioides difficile* infection

**DOI:** 10.1017/ash.2024.454

**Published:** 2024-11-18

**Authors:** Shelley E. Kon, Christine Gunter, Heidi Hough, Randi Craig, Ashley M. Lane, Mary T. Bessesen

**Affiliations:** 1 VA Eastern Colorado Health Care System, Aurora, CO, USA; 2 University of Colorado School of Medicine, Aurora, CO, USA

## Abstract

**Objective:**

To develop a symptom assessment tool to assist health care providers with discussing bowel habits in a sensitive and accurate method.

**Design:**

Pre and Post education survey.

**Setting:**

180 bed academically affiliated Veterans Affairs Hospital.

**Participants:**

Nurses, nursing assistants and physicians who participated in a brief educational session.

**Methods:**

A Bowel Habits Assessment Tool (BHAT) was developed to assist health care providers in learning skills to assess patient bowel habits accurately. The BHAT was introduced at educational sessions. An anonymous pre and post survey employing a 5-point Likert scale was administered to participants.

**Results:**

Pre-educational session survey results for Question 1 (Q1) “I am comfortable discussing patient’s bowel habits included: 4.6% strongly disagreed or somewhat disagreed, 3% neither agreed nor disagreed, 20% somewhat agreed and 72.3% strongly agreed. After the BHAT education, 100% (n=65) of participants strongly agreed or somewhat agreed that they were comfortable discussing patient’s bowel habits. Q1 pre/post mean difference 0.25 (CI 0.06539 - 0.4269, p = 0.0084). On the pre-survey, only 34% of participants strongly agreed that they were aware of tools to help discuss patients’ bowel habits. This increased to 77% after the BHAT educational session. (Q2). Q2 pre/post mean difference 1.02 (CI 0.7366 - 1.294, p <0.0001).

**Conclusions:**

The BHAT improved clinicians’ comfort level discussing patient’s bowel habits. Health care providers found the BHAT useful and related to their work. This tool shows promise in improving providers’ comfort discussing bowel habits and diagnosing *Clostridioides difficile* in a timely manner.

## Introduction

*Clostridioides difficile* infection (CDI) affects approximately 500,000 patients annually in the United States. CDI symptoms range from mild diarrhea to life threatening colitis and death.^1,2^ Hospital onset (HO)-CDI has significant associated morbidity and mortality. One in 11 people over age 65 diagnosed with HO-CDI will die within one month.^3^ NSHN defines HO-CDI as a positive *C.difficile* test collected from an inpatient on or after hospital day four.^4^ At our facility, we noted that several patients with CDI had symptoms on admission, but were not tested until after hospital day three, and therefore were misclassified as HO-CDI. This is supported by the literature, as Nanayakkara et. al noted that 20% of their community onset (CO) CDI cases were misclassified as HO-CDI.^5^ Timely diagnosis of CDI is important for surveillance classification and for timely initiation of isolation and treatment.

Previously, our team utilized a novel humble inquiry interview method to discuss CDI with nursing staff and inquire why there may be a delay in CDI diagnosis. Our nursing staff noted that patients may be reluctant to discuss their bowel habits.^6^ Prior literature supports the observation that discussing bowel habits can be a socially taboo subject.^7^ This social taboo can prevent patients disclosing GI issues to their providers.^8^ For example, studies have shown that there is stigma around GI conditions such as irritable bowel syndrome, diarrhea and fecal incontinence among patients and providers^8,9^.

Therefore, we hypothesized that a symptom assessment tool may increase healthcare workers’ comfort discussing bowel habits, be useful and related to clinicians’ work, ultimately leading to more timely diagnosis of CDI and other gastrointestinal conditions.

## Methods

A Bowel Habits Assessment Tool (BHAT) was developed to assist healthcare providers in assessing patient bowel habits accurately, providing a structured approach to gather relevant information, identify abnormalities, and promote effective communication with patients (See Table 1). The tool was developed by a team of infectious disease physicians, nurses and a gastroenterologist in June 2023 and modeled on existing tools utilized to take a sexual history. The tool was piloted at a 180 bed academically affiliated Veterans Affairs Hospital. Small group sessions held periodically over a seven-month period (November 2023–May 2024) provided education about the importance of a bowel habit history and reviewed the tool. An infectious disease physician and a nurse infection preventionist led the teaching sessions. An anonymous pre- and postsurvey employing a 5-point Likert scale was administered to participants. The presurvey included the statements “I am comfortable discussing patient’s bowel habits” (Question 1, Q1), “I am aware of tools to help discuss patients bowel habits” (Question 2, Q2) and “How often do you discuss bowel habits” (Question 3, Q3). The postsurvey included the same questions with two additional questions evaluating the tool. Paired t tests were employed using GraphPad Prism® 10.1.2 (324). All participation was voluntary. This project was approved as a Quality Improvement project by the VA Research Service, Eastern Colorado Health Care System.

**Table 1.** Outline of the Bowel Habit Assessment Tool (BHAT). The BHAT is a 3-page guide. The introduction states that bowel habits can be a taboo subject and highlights the importance of an accurate bowel habit history. The step-by-step guide details a suggested dialogue with the patient. A picture of the Bristol Stool scale is provided for reference. The complete tool is available in the supplemental material

Outline of the Bowel Habit Assessment Tool (BHAT)	
Section:	Description:
1. Introduction	Provides information about why the tool is necessary, an outline of the tool and how it will benefit clinicians and patients.
2. Set the stage	Discusses the importance of making the patient comfortable and having the conversation in a quiet private room.
3. Step-by step Assessment Guide	Provides a structured dialogue with the patient: 1. May I ask you some personal questions about your bowel habits? I understand these questions are not often discussed, but they are important for your overall health. 2. Start with an open-ended question. Can you tell me about your normal bowel movements? 3. How many bowel movements have you had in the last 24 hours? 4. Gather specific information: When was you last bowel movement? 5. How often are you having bowel movements? (frequency). 6.What do they look like? a. You can use words like hard, lumpy, sausage shaped, smooth, soft, mushy or entirely liquid? b. Reference the Bristol Stool Chart if needed! 7. Any associated symptoms? 8. Red flags: Any black tarry stools or blood in the stool?
4. Bristol Stool Scale	Picture of the Bristol Stool Scale.
5. Quick Tips	Summarizes the tool in quick easy steps.

## Results

Sixty-eight healthcare personnel participated in the pilot, nurses (39), resident physicians (19), nursing assistants/healthcare technicians (4), and 6 others (medical students, nursing students, unknown and administrator). Three surveys were excluded, two incomplete and one completed by an administrator.

Prior to the educational session, 72.3% (n = 47) of participants strongly agreed with the statement “I am comfortable discussing patient’s bowel habits” (Q1). This increased to 83% (n = 50) after the BHAT educational session. 7.7% (n = 5) participants chose neither disagree or agree, somewhat disagree or strongly disagree for Q1. After the education, no participants disagreed and 100% (n = 65) of included participants strongly agreed or somewhat agreed they were comfortable discussing patient’s bowel habits. The mean difference in the Likert scale scores between pre- and postsurvey responses for Q1 was 0.25 (CI 0.06539–0.4269, *P* = 0.0084).

On the presurvey, only 34% of participants strongly agreed that they were aware of tools to help discuss patients’ bowel habits (Q2). This increased to 77% after the BHAT educational session. The mean difference in the Likert scale score between pre- and postsurvey responses for Q2 was 1.02 (CI 0.7366–1.294, *P* < 0.0001). When asked to describe how often they discuss bowel habits with patients, 42.2% (n = 27) of responding participants selected “only when needed,” 32.8% (n = 21) of participants chose “every shift,” 12.5% (n = 8) of participants chose “every visit” and 12.5% (n = 8) chose “every day.” One participant (n = 1) left this question blank. 98.5% (n = 64) of participants either strongly or somewhat agreed that the BHAT was related to their work. Majority of participants found the BHAT useful, with 66.2% strongly agreeing and 30.8% (n = 20) somewhat agreeing that the BHAT was useful.

## Discussion

Although there are existing patient questionnaires related to bowel habits, we are not aware of any existing tool specifically focusing on communication with patients. The 34% of participants who were aware of tools prior to the educational sessions may have been referring to existing patient directed questionnaires or alternatively heard about the tool from other staff. The BHAT shows promise in improving providers’ comfort discussing bowel habits. Improved comfort may lead to decreased stigma and patients disclosing GI symptoms more readily. Future directions include updating the tool based on feedback, soliciting patient feedback and potentially advising more frequent assessment of bowel habits. If hospitalized patients are more willing to disclose symptoms of diarrhea, then providers can test for CDI or investigate other undiagnosed GI conditions in a timely manner.

## Supporting information

Kon et al. supplementary material Kon et al. supplementary material

## Data Availability

Full data is available upon request by contacting the corresponding author.

## Supplementary material

For supplementary material accompanying this paper visit http://doi.org/10.1017/ash.2024.454. click here to view supplementary material
