# Supplementary material for: Pilot of a bowel habit assessment tool to enable early identification of Clostridioides difficile infection
Source: Antimicrob Steward Healthc Epidemiol. 2024 Nov 18;4(1):e208. doi: 10.1017/ash.2024.454 (PMC11574595; doi:10.1017/ash.2024.454)
Supplement: Kon et al. supplementary material [file S2732494X24004546sup001.pdf]

Bowel Habits **Assessment Tool**

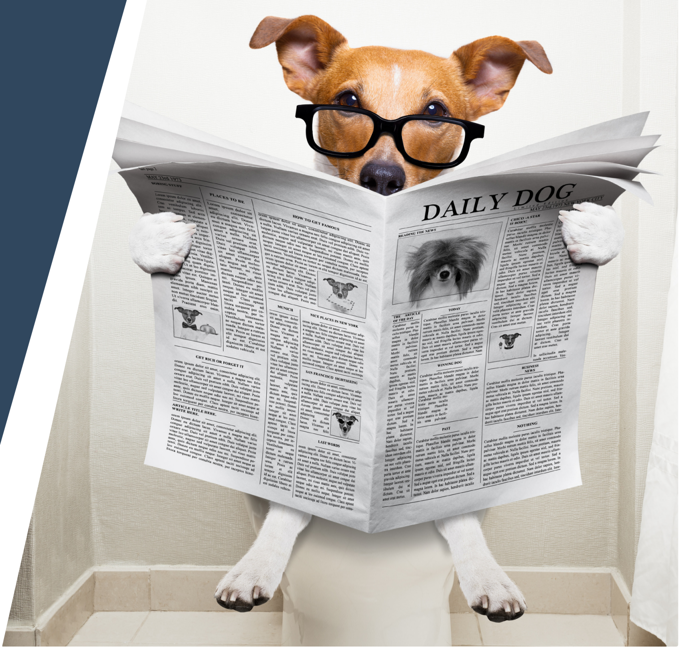

Introduction

Pooping is a part of life, but it can often be a taboo subject. Gastrointestinal conditions are common in hospitalized patients. Patient's bowel movements may be altered by lack of mobility, new medications and their underlying illness.

It's important for health care providers to be aware of patient's bowel habits. In order for patients to get the best care, we must ask them about their bowel habits and document them accurately. Patient modesty may be a barrier to discussing their bowel habits with nurses and providers. This can lead to delay in diagnosis of important issues such as constipation, gastrointestinal bleeding and Clostridiodes difficile infection (CDI).

This **Bowel Habits Assessment Tool (BHAT)** is designed to assist health care providers in developing the necessary skills to assess and document patient bowel habits accurately.

- This tool will provide a structured approach to help clinicians gather relevant information, identify abnormalities, and promote effective communication with patients.
- By using this training tool, clinicians can enhance their ability to provide optimal care and support to patients with bowel-related concerns.
- This tool is not designed to be a comprehensive gastrointestinal clinical history but focuses more on developing communication skills to address a sensitive subject.

Resources:

https://www.cdc.gov/nchs/fastats/digestive-diseases.htm

https://www.cdc.gov/cdiff/index.html

INFECTION PREVENTION RMR VA

1. Set the Stage

Some patients may not be comfortable talking about their bowel habits. Make sure to have the conversation in a quiet private room. Try to put patients at ease by emphasizing that stool and defecation are a normal part of life.

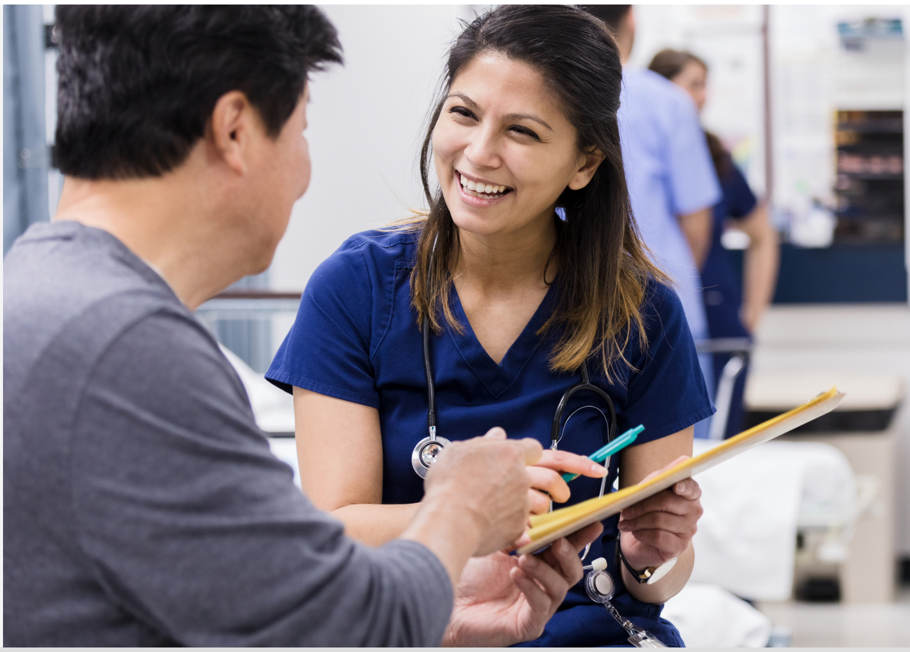

2. Step-by-Step Assessment Guide

Dialogue with the Patient

1. May I ask you some personal questions about your bowel habits? I understand these questions are not often discussed, but they are important for your overall health.

2. Start with an open ended question. Can you tell me about your normal bowel movements?

3. How many bowel movements have you had in the last 24 hours?

4. If no BM in the last 24 hours, ask: When was you last bowel movement?

5. What do they look like?

a. You can use words like hard, lumpy, sausage shaped, smooth, soft, mushy or entirely liquid?

b. Reference the Bristol Stool Chart if needed!

6. Any associated symptoms?

7. Red flags: Any black tarry stools or blood in the stool?

Bristol Stool Scale

Type 1	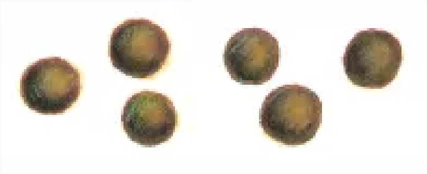	Separate hard lumps, like nuts (hard to pass)
Type 2	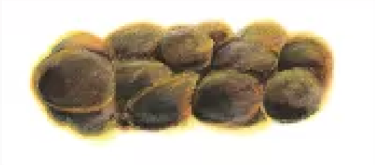	Sausage-shaped but lumpy
Type 3	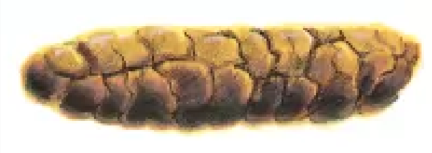	Like a sausage but with cracks on its surface
Type 4	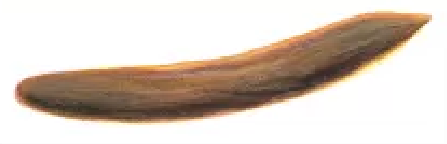	Like a sausage or snake, smooth and soft
Type 5	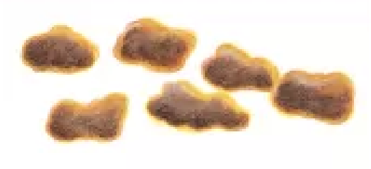	Soft blobs with clear-cut edges (passed easily)
Type 6	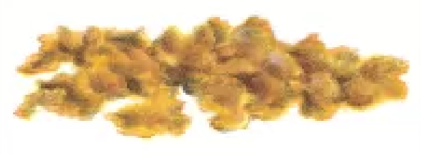	Fluffy pieces with ragged edges, a mushy stool
Type 7	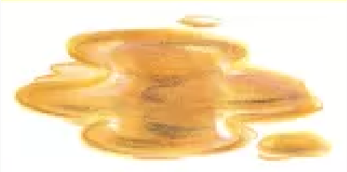	Watery, no solid pieces ENTIRELY LIQUID

Reference: Heaton, K W & Lewis, S J 1997, 'Stool form scale as a useful guide to intestinal transit time'. Scandinavian Journal of Gastroenterology, vol.32, no.9, pp.920 - 924

Quick Tips for addressing bowel habits:

Ask.
Acknowledge that discussing poop can be uncomfortable. Establish baseline and changes.
Check frequency, consistency.
Assess for red flags (blood in stool).
